# Accuracy of salivary biomarkers in the diagnosis of periodontal status and coronary heart disease

**DOI:** 10.25122/jml-2023-0264

**Published:** 2024-04

**Authors:** Zina Ali Daily, Batool Hassan Al-Ghurabi

**Affiliations:** 1 Department of Periodontics, College of Dentistry, University of Al-Ameed, Karbala, Iraq; 2 Department of Periodontics, College of Dentistry, University of Baghdad, Baghdad, Iraq; 3 Department of Basic Science, College of Dentistry, University of Baghdad, Baghdad, Iraq

**Keywords:** periodontitis, atherosclerotic coronary heart disease, IL-1β, IL-18, gasdermin D, AUC, area under the curve, CAL, clinical attachment level, CHD, coronary heart disease, CT, computed tomography, HDL, high-density lipoprotein, ICC, intraclass correlation coefficient, LDL, low-density lipoprotein, PLI, plaque index, PPD, pocket probing depth, TC, total cholesterol, TG, triglyceride

## Abstract

Inflammatory illnesses, such as periodontitis and atherosclerotic coronary heart disease (ASCHD), trigger the production of pro-inflammatory mediators. The aim of this study was to assess the accuracy of using salivary interleukin-1β (IL-1β), interleukin-18 (IL-18), and gasdermin D (GSDMD) in discerning patients with periodontitis with and without ASCHD from healthy individuals, and to assess their correlation with clinical periodontal parameters and low-density lipoprotein (LDL) levels. The study involved 120 participants: 30 were healthy subjects (control group, C), 30 had generalized periodontitis (group P), 30 had ASCHD and clinically healthy periodontium (group AS-C), and 30 had ASCHD and generalized periodontitis (group AS-P). Saliva and blood samples were collected, and periodontal characteristics such as plaque index, bleeding on probing, probing pocket depth, and clinical attachment loss were examined. IL-1β, IL-18, and GSDMD levels from saliva were determined using ELISA. LDL levels were determined from the blood samples. Groups P, AS-C, and AS-P had higher levels of salivary IL-1β, IL-18, and GSDMD than group C. The receiver operating characteristic (ROC) curves of all biomarkers showed high diagnostic accuracy, with a significant positive correlation with the clinical parameters and LDL levels. The observed correlations between the studied pro-inflammatory mediators and disease severity suggest that these biomarkers could serve as indicators of disease progression in conditions such as periodontitis and ASCHD.

## INTRODUCTION

Periodontitis, an infection-based inflammatory disease, destroys the alveolar bone and connective tissues that keep teeth in place. Pathobionts cause tissue damage by switching from a symbiotic to dysbiotic relationship [1,2]. The dysbiotic flora affects immunological responses to bacterial infection, affecting disease distribution and severity [3].

Atherosclerosis (AS) is an inflammatory disease that develops when the immune system initiates, spreads, and activates lesions across the cardiovascular system in response to an increase in circulating low-density lipoproteins (LDLs) and cholesterol. Inflammation plays a key role in coronary heart disease (CHD) and other manifestations of atherosclerosis. However, the correlation between atherosclerotic coronary heart disease (ASCHD) and periodontitis is not yet fully understood [4,5].

Research indicates that toll-like receptor signaling pathways and inflammasome complexes form in response to either exogenous or sterile signals. The autoproteolytic maturation of caspase-1 triggers the production of interleukin-1β (IL-1β), interleukin-18 (IL-18), and gasdermin D (GSDMD) N-terminal. This process forms a membrane pore through the cleaving of N-GSDMD fragments, leading to the release of inflammatory cytokines, such as IL-1β and IL-18, into the extracellular space during pyroptosis. These inflammatory processes are associated with periodontal soft tissue damage, osteoclastogenesis, and atherogenesis [6–8].

The host response in periodontal disease and CHD is characterized by the production of key pro-inflammatory cytokines, such as IL-1β, IL-18, and GSDMD [9,10]. Although previous studies have examined the role of IL-18 and GSDMD in the destruction of periodontal tissue and in atherosclerosis [11,12], data is scarce regarding the salivary levels of IL-1β, IL-18, and GSDMD in patients with periodontitis, with and without CHD. The aim of this study was to assess the accuracy of salivary IL-1β, IL-18, and GSDMD levels in differentiating patients with periodontitis with and without ASCHD from clinically healthy individuals, and to examine the correlation of these potential biomarkers with clinical periodontal parameters and LDL levels.

## MATERIALS AND METHODS

### Study design

We conducted a retrospective case-control study at the Department of Periodontics of Al-Ameed University Dentistry College and Hospital, the Karbala Specialized Dental Centre, the Imam Al-Hussain Medical City, and the Karbala Centre of Cardiovascular Diseases and Surgery between April and October 2022. The study was approved by the ethics committee of the College of Dentistry at University of Baghdad (approval no. 652622). Each participant was provided detailed information about the study and its purposes, and signed an informed consent. The inclusion criteria were willingness to participate in the study, possessing ≥20 healthy teeth, and being in good overall health, with no other systemic comorbidities apart from AS diagnosed through cardiac catheterization. The exclusion criteria included systemic diseases that may affect the progression of periodontitis, periodontal treatment in the last 6 months, smoking or other vices, corticosteroid or antibiotic treatment in the last 3 months, and pregnancy or nursing at the time of the study. Based on the reported prevalence of periodontitis and CHD, a number of 120 participants (30 subjects per group) was determined to be sufficient, with a test power of 80% and an α probability of 0.05 [13].

### Participant profiles

The study included 120 patients, 95 men and 25 women aged between 35 and 75 years. All patients had a body mass index (BMI) of <25 kg/m^2^. Based on the reported prevalence of periodontitis and CHD, a number of 83 patients were determined to be sufficient with a test power of 80% and an α probability of 0.05 [13]. This number was increased to 90 to calculate for potential dropouts. The subjects were divided into three groups: 30 subjects with periodontitis (group P), 30 subjects with periodontitis and CHD (group AS-P), and 30 subjects with CHD (group AS-C). Another 30 healthy subjects were used as controls.

First, the participants were examined visually to ensure that subjects with periodontitis (groups P and AS-P) had an interdental clinical attachment loss (CAL) of ≥2 non-adjacent teeth or CAL of ≥3 mm on the buccal (facial) or lingual/palatal aspects, a probing pocket depth (PPD) of >3 mm at more than two teeth, periodontitis stage III or IV, grade B or C with unstable cases, meaning a PPD of ≥4 mm with bleeding on probing (BOP) or PPD of >5 mm with or without BOP [14,15]. Patients with CHD (groups AS-P and AS-C) had to have been diagnosed with clinical dyspnea; chest pain; electrocardiogram (ECG) changes; laboratory-confirmed elevated LDL, total cholesterol (TC), and triglyceride (TG) levels; and laboratory-confirmed low high-density lipoprotein (HDL) levels. Patients with CHD were diagnosed by a cardiologist using noninvasive screening tests such as coronary computed tomography (CT) angiography, and diagnostic cardiac catheterization procedure in case of atherosclerotic plaque lesions of >50% [16–19]. Healthy subjects (group C) had clinically healthy periodontium, BOP < 10%, PPD ≤ 3 mm, and intact periodontium [15].

### Examination of periodontal parameters

The periodontal parameters of each tooth were clinically evaluated, including the full-mouth plaque index (PLI) [20], full-mouth BOP [21], PPD, and CAL. A Hu-Friedy University of North Carolina 15 probe was used for the assessment. The percentage of bleeding was recorded at six sites per tooth (mesiofacial, facial, distofacial, mesiolingual, lingual, and distolingual), and PPD and CAL were recorded to the nearest millimeter. The visually determined percentages of O’Leary PLI scores at four surfaces were also recorded. The wisdom teeth were not examined.

### Saliva sample collection and analysis

Before saliva collection, the participants were encouraged to brush their teeth and floss. Saliva samples were collected via passive drooling into clean plastic cups between 09:00 and 12:00. A micropipette was then used to transfer 300 µl of the saliva sample into an Eppendorf tube (Thermo Fisher Scientific), which was then centrifuged at 3,000×*g* for 20 min and stored at −20 °C. The protein concentration of each biomarker was measured using ELISA. Commercially available ELISA kits (My BioSource) were used to determine the levels of IL-1β, IL-18, and GSDMD in the saliva according to the manufacturer’s instructions. The absorbance of all proteins was measured using a spectrophotometer plate reader. The detection limits for IL-1β, IL-18, and GSDMD were 2.0 pg/ml, 1.5 pg/ml, and 0.5 pg/ml, respectively. The sensitivity and specificity of the biomarkers were determined using a receiver operating characteristic (ROC) curve and the area under the curve (AUC). The sandwich method was used to prevent cross-reactivity, and involved using a plate pre-coated with human-specific antibodies. This ensured that specific proteins in the sample bound exclusively to the antibodies coating the wells. In addition, biotinylated human-specific antibodies were added to bind specifically to these proteins.

The reliability of the study was ensured through the analysis of intraexaminer reliability. This involved conducting calibration sessions for the assessed clinical periodontal parameters twice within an hour, and the examination of five patients with generalized periodontitis not included in the study. The intraclass correlation coefficient (ICC) was 0.94 for PPD and 0.88 for CAL, whereas the average kappa (*Κ*) coefficients were 0.89 for BOP and 0.91 for PLI. Therefore, the reliability of the study was satisfactory.

### Blood sample collection

Approximately 2 ml of peripheral blood were collected and placed within 30 s in a tube containing ethylene diamine tetra acetic acid (EDTA). The blood samples were then analyzed in a laboratory to determine the LDL levels.

### Statistical analysis

Statistical analyses were performed using SPSS v.22 (IBM Corp). For descriptive statistics, all the data was expressed as mean ± s.d. and medians. The Gaussian distribution of the data was determined using the Shapiro–Wilk test, which indicated that data obtained from ELISA were normally distributed. Each study group’s parameters were expressed as mean ± s.d., and compared using one-way analysis of variance (ANOVA) followed by a post-hoc Tukey test. The chi-squared test was used to compare participant data by sex. The Pearson correlation test was used to assess the correlation between the analyzed clinical variables analyzed and LDL versus salivary IL-1β, IL-18, and GSDMD levels, as well as among the salivary levels of IL-1β, IL-18, and GSDMD. To evaluate the accuracy of using IL-1β, IL-18, and GSDMD levels to differentiate between healthy individuals and patients with severe periodontitis with and without ASCHD, and to distinguish between periodontitis and AS-C, AS-P, as well as between AS-C and AS-P, ROC curves were constructed. The AUC was calculated to identify cut-off points and estimate the sensitivity and specificity, with a 95% confidence interval (CI). Statistical significance was considered at *P* ≤ 0.05.

## RESULTS

There were no statistically significant differences between the four groups regarding participant age, sex, and BMI (*P* > 0.05). The analysis of periodontal parameters revealed a statistically significant increase of the mean PLI% and mean BOP% in all four groups and of the mean PPD and mean CAL in groups P and AS-P. Significantly higher levels of mean LDL were observed in groups AS-C and AS-P.

**Figure 1:**
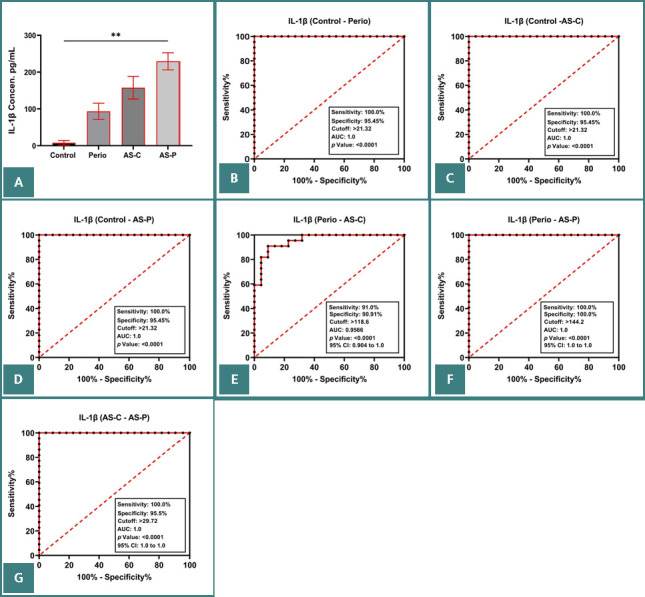
A, Mean salivary IL-1β levels in the studied groups. B–G, Logistic regression analysis in group C vs. group P (B), group C vs. group AS-C (C), group C vs. group AS-P (D), group P vs. group AS-C (E), group P vs. group AS-P (F), and group AS-C vs. group AS-P (G).

**Figure 2:**
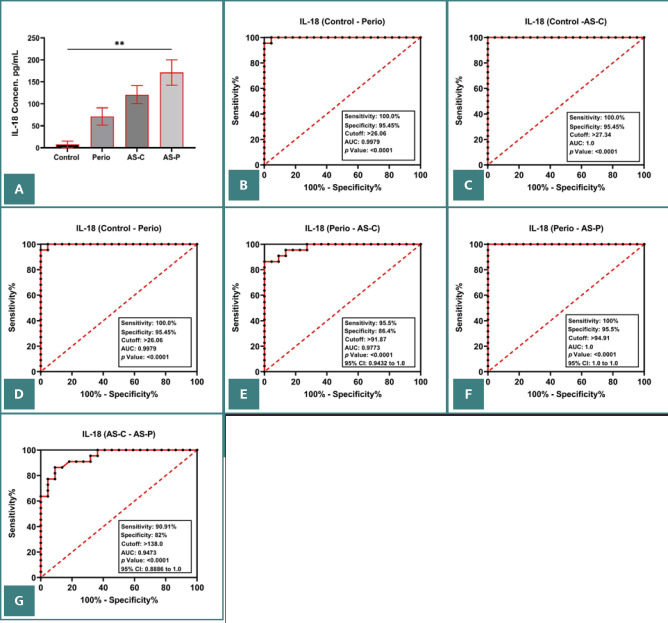
A, Mean salivary IL-18 levels in the studied groups. B–G, Logistic regression analysis in in group C vs. group P (B), group C vs. group AS-C (C), group C vs. group AS-P (D), group P vs. group AS-C (E), group P vs. group AS-P (F), and group AS-C vs. group AS-P (G).

**Figure 3:**
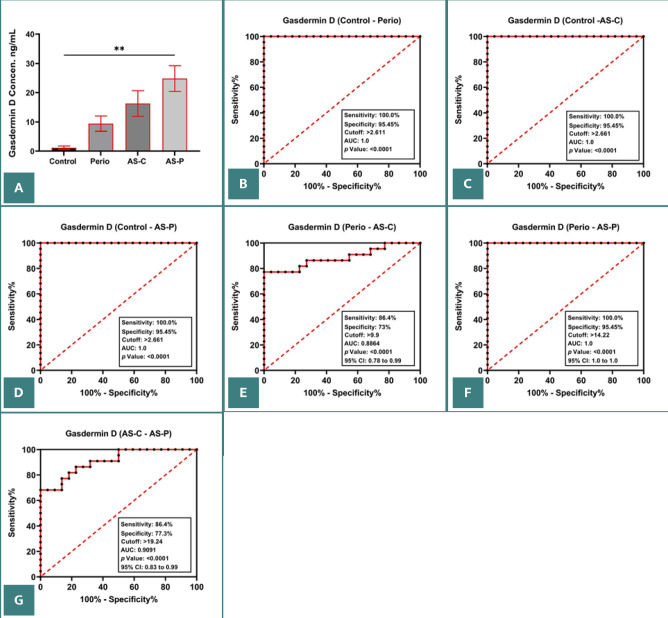
Mean salivary GSDMD levels in the studied groups. B–G, Logistic regression analysis in in group C vs. group P (B), group C vs. group AS-C (C), group C vs. group AS-P (D), group P vs. group AS-C (E), group P vs. group AS-P (F), and group AS-C vs. group AS-P (G).

Furthermore, mean salivary IL-1β levels were significantly higher in groups P (78.43 pg/ml), AS-C (123.29 pg/ml), and AS-P (194.15 pg/ml) than in group C (5.94 pg/ml) (*P* < 0.001; Figure 1A). Mean salivary IL-18 levels were significantly higher in groups P (65.862 pg/ml), AS-C (102.418 pg/ml), and AS-P (145.773 pg/ml) than in group C (6.594 pg/ml) (*P* < 0.001; Figure 2A and Table 1). Similarly, mean salivary GSDMD levels were significantly higher in groups P (8.647 pg/ml), AS-C (14.772 pg/ml), and AS-P (20.894 pg/ml) than in group C (1.019 pg/ml) (*P* < 0.001; Figure 3A and Table 1).

**Table 1:** The demographic characteristics, clinical periodontal parameters, IL-1β, 1L-18, GSDMD, and LDL levels of the studied groups. Data are expressed as mean ± s.d.

Variable	Control (C) (*n* = 30)	Generalized periodontitis (P) (*n* = 30)	CHD and clinically healthy periodontium (AS-C) (*n* = 30)	CHD with general periodontitis (AS-P) (*n* = 30)	*P* value
Age (years)	54.03 ± 7.62	54.83 ± 7.5	58.06 ± 7.00	57.76 ± 5.91	0.061
BMI	21.35 ± 1.58	21.17 ± 1.27	21.79 ± 1.44	21.42 ± 2.17	0.529
Sex					
Male (**n**, %)	24 (80.0%)	24 (80.0%)	24 (80.0%)	23 (76.7%)	1.00
Female (**n**, %)	6 (20.0%)	6 (20.0%)	6 (20.0%)	7 (23.3%)	
PLI (%)	11.799 ± 2.297	65.102 ± 1.193	18.414 ± 2.702	68.691 ± 2.385	<0.001^*^
BOP (%)	5.558 ± 1.547	50.940 ± 2.989	5.749 ± 1.696	52.353 ± 2.236	<0.001^*^
PPD (mm)	0.00 ± 0.00	4.876 ± 1.599	0.00 ± 0.00	7.228 ± 1.740	<0.001^*^
CAL (mm)	0.00 ± 0.00	6.563 ± 1.603	0.00 ± 0.00	8.001 ± 1.500	<0.001^*^
IL-1β (pg/ml)	5.940 ± 1.735	78.43 ± 12.089	123.289 ± 22.448	194.145 ± 27.125	<0.001^*^
IL-18 (pg/ml)	6.594 ± 2.937	65.862 ± 13.96	102.418 ± 8.47	145.77 ± 6.29	<0.001^*^
Gasdermin D (pg/ml)	1.019 ± 0.195	8.647 ± 0.989	14.772 ± 1.773	20.894 ± 3.077	<0.001^*^
LDL (mg/dl)	41.37 ± 5.83	48.54 ± 8.23	119.23 ± 12.82	148.28 ± 21.43	<0.001^*^

* Statistically significant

The ROC curves indicated a high accuracy of salivary IL-1β, IL-18, and GSDMD levels as biomarkers of periodontitis and CHD (Figures 1B–G, 2B–G, and 3B–G), with an AUC of 1.0 for groups P, AS-C, and AS-P (*P* < 0.0001). This indicated a sensitivity of 100.00% and a specificity of 95.45% at cut-off points of 21.32, 27.34, and 2.661 for groups P, AS-C, and AS-P, respectively. Then, group P was compared with groups AS-C and AS-P, and group AS-C was compared with group AS-P (Figures 1D–G, 2D–G, and 3D–G).

There was a significant positive correlation between IL-1β levels and BOP (*r =* 0.469, *P* = 0.028), as well as IL-1β levels and PPD (*r =* 0.570, *P* = 0.005) in group P; between IL-1β levels and BOP (*r =* 0.361, *P* = 0.048), as well as IL-1β levels and CAL (*r =* 0.544, *P* = 0.009) in group AS-P; between IL-18 levels and PPD (*r =* 0.463, *P* = 0.030), as well as between IL-18 levels and CAL in group P (*r =* 0.364, *P* = 0.048) and in group AS-P (*r =* 0.586, *P* = 0.004); as well as between GSDMD levels and CAL in groups P (*r =* 0.395, *P* = 0.031) and AS-P (*r =* 0.503, *P* = 0.017). There was also a significant positive correlation between IL-1β levels and LDL levels in groups AS-C (*r =* 0.462, *P* = 0.028) and AS-P (*r =* 0.499, *P* = 0.020); between IL-18 levels and LDL levels in groups AS-C (*r =* 0.432, *P* = 0.016) and AS-P (*r =* 0.477, *P* = 0.008); and between GSDMD levels and LDL levels in groups AS-C (*r =* 0.335, *P* = 0.040) and AS-P (*r =* 0.394, *P* = 0.031) (Table 2).

**Table 2:** Pearson correlation coefficients (**r**) between salivary IL-1β, IL-18, and GSDMD levels with clinical periodontal parameters and LDL levels

Biomarkers		PLI		BOP		PPD		CAL		LDL	
	Patient group	*r*	*P* value	*r*	*P* value	*r*	*P* value	*r*	*P* value	*r*	*P* value
IL-1β	C	0.007	0.975	0.188	0.402	0.00	0.00	0.00	0.00	0.262	0.238
	P	0.062	0.785	0.469	0.028	0.570	0.005	0.070	0.756	0.297	0.179
	AS-C	0.009	0.969	0.048	0.831	0.00	0.00	0.00	0.00	0.462	0.028^*^
	AS-P	0.009	0.968	0.361	0.048	0.329	0.536	0.544	0.009	0.499	0.020^*^
IL-18	C	0.090	0.691	0.141	0.532	0.00	0.00	0.00	0.00	0.241	0.187
	P	0.206	0.357	0.272	0.221	0.463	0.030	0.364	0.048	0.272	0.147
	AS-C	0.056	0.806	0.186	0.407	0.00	0.00	0.00	0.00	0.432	0.016^*^
	AS-P	0.030	0.895	0.011	0.961	0.374	0.218	0.586	0.004	0.477	0.011^*^
GSDMD	C	0.193	0.389	0.183	0.415	0.00	0.00	0.00	0.00	0.195	0.302
	P	0.220	0.325	0.193	0.934	0.229	0.923	0.395	0.031	0.293	0.116
	AS-C	0.071	0.755	0.091	0.689	0.00	0.00	0.00	0.00	0.335	0.040^*^
	AS-P	0.075	0.740	0.220	0.324	0.404	0.362	0.503	0.017	0.394	0.031^*^

* Statistically significant

There was a significant positive correlation between IL-18 and GSDMD levels in group P (*r =* 0.618, *P* = 0.002), between IL-18 and IL-1β levels in group AS-C (*r =* 0.503, *P* = 0.017), and between IL-1β and GSDMD levels in group AS-P (*r =* 0.437, *P* = 0.021), and between IL-1β and IL-18 levels in group AS-P (*r =* 0.592, *P* = 0.003) (Table 3).

**Table 3:** Pearson correlation coefficients (**r**) between salivary IL-1β, IL-18, and GSDMD levels

Patient group		IL-18		IL-1β	
		*r*	*P* value	*r*	*P* value
C	GSDMD	0.005	0.986	−0.051	0.822
	IL-18	–	–	−0.090	0.690
P	GSDMD	0.618	0.002^*^	0.111	0.624
	IL-18	–	–	−0.047	0.837
AS-C	GSDMD	−0.254	0.253	−0.186	0.407
	IL-18	–	–	0.503	0.017^*^
AS-P	GSDMD	−0.289	0.193	0.437	0.021^*^
	IL-18	–	–	0.592	0.003^*^

* Statistically significant

## DISCUSSION

This study found that groups P, AS-C, and AS-P exhibited significantly higher levels of IL-1ß, IL-18, and GSDMD than group C. Furthermore, all the biomarkers showed high accuracy in differentiating patients with periodontitis with and without ASCHD from healthy individuals. The biomarkers also showed a significantly positive correlation with higher clinical periodontal parameters and LDL levels.

Groups P, AS-C, and AS-P exhibited higher levels of salivary IL-1β than group C. This suggests a potential correlation between periodontal tissue destruction, atherogenesis, and elevated salivary IL-1β levels. The increase in IL-1β levels may result from stimulation by microbial components and oxidized LDL, and has an important role in directly inducing osteoclastogenesis, substantial periodontium destruction, and endothelial cell dysfunction [22–24]. Consequently, IL-1β is a salivary pro-inflammatory cytokine that could potentially indicate current disease activity, severity, and progression for periodontitis and CHD. Similarly to these results, studies by Al-Taweel *et al*. [16] and Gita *et al*. [17] found that IL-1β levels could be used to differentiate between healthy individuals and those with severe periodontitis and ASCHD, demonstrating high accuracy based on ROC curve analysis and superior performance in patient groups.

We also found that salivary IL-18 levels were significantly higher in groups P, AS-C, and AS-P than in group C. This suggests that IL-18 may have a regulatory role in inflammation and tissue damage, acting both as a pro-inflammatory and anti-inflammatory cytokine. Elevated IL-18 levels have been linked to a decline in periodontal health and dysfunction of blood vessel cells. Studies by Zhang *et al*. [25] and Jefferis *et al*. [11] highlighted the pleiotropic functions of IL-18 and the detrimental effects of excessive IL-18 levels on bone and soft tissues in the periodontium, including blood vessel cell dysfunction, inflammation, and the development of atherosclerotic plaques. They also found that IL-18 levels can be used to differentiate between healthy individuals and patients with severe periodontitis and ASCHD.

Salivary GSDMD levels were also significantly higher in groups P, AS-C, and AS-P than in group C. This suggests that GSDMD is a proinflammatory pore-forming mediator that has a vital regulatory role in inflammation and tissue damage through pyroptosis. Periodontal disease, endothelial cell malfunction, and cardiomyocyte pyroptosis have been associated with higher levels of GSDMD. Studies by Zhuang *et al*. [26] and Weng *et al*. [12] found that the activation of GSDMD altered the pyroptotic rate of human periodontal ligament cells, leading to the formation and release of IL-1 and IL-18. In addition, Shi *et al*. [27] found that higher levels of circulating GSDMD were the result of hypoxia and reoxygenation of cardiomyocytes that had undergone pyroptosis. The analysis of ROC curves revealed that GSDMD levels can be used to accurately differentiate between healthy individuals, patients with severe periodontitis, and patients with ASCHD.

Periodontal parameters assessed in this study, including PLI, BOP, PPD, and CAL, were found to be highest in group AS-P. Al-Taweel *et al*. [16], Yagnik *et al*. [18], Al-Ghurabi *et al*. [28], and Shaker and Hashem [29] reported similar findings and concluded that the inflammatory response could be attributed to the formation of plaque biofilms, which produce an extensive variety of bacterial byproducts. Our study revealed that patients with ASCHD have significantly higher levels of LDL than patients with periodontitis and healthy individuals. This may be attributed to the involvement of adipocytes and elevated levels of LDL, TC, and TG in the pathogenesis of inflammatory diseases such as ASCHD, leading to the production of pro-inflammatory cytokines [30].

The findings of this study indicate that there is a significant positive correlation between salivary IL-1β, IL-18, and GSDMD levels and clinical periodontal parameters in patients with periodontitis, as well as LDL levels in patients with ASCHD. These correlation can be attributed to the triggering of inflammatory pathways and the excessive production of pro-inflammatory mediators in response to persistent etiologic factors. The present study also determined that salivary IL-1β, IL-18, and GSDMD positively and significantly correlate between groups P, AS-C, and AS-P.

## CONCLUSION

The observed correlations between the studied pro-inflammatory mediators and disease severity suggest that these biomarkers could serve as indicators of disease progression in conditions such as periodontitis and ASCHD.

## Data Availability

The data that support the findings of this study are available on request from the corresponding author. The data are not publicly available due to privacy or ethical restrictions.

## References

[1] Orlandi M, Graziani F, D’Aiuto F (2020). Periodontal therapy and cardiovascular risk. Periodontol 2000.

[2] Abdulkareem AA, Abdulbaq HR, Milward MR (2020). In Vitro Homeostasis of Rat Oral Epithelial Cell Cultures Following Withdrawal of Periodontal Pathogens. Braz Dent J.

[3] Laine ML, Crielaard W, Loos BG (2012). Genetic susceptibility to periodontitis. Periodontol 2000.

[4] Herrera D, Molina A, Buhlin K, Klinge B (2020). Periodontal diseases and association with atherosclerotic Disease. Periodontol 2000.

[5] Zardawi F, Gul S, Abdulkareem A, Sha A, Yates J (2021). Association Between Periodontal Disease and Atherosclerotic Cardiovascular Diseases: Revisited. Front Cardiovasc Med.

[6] Al-Obaidi MJ, Al-Ghurabi BH (2023). Potential Role of NLRP3 Inflammasome Activation in the Pathogenesis of Periodontitis Patients with Type 2 Diabetes Mellitus. Med Chem Sci.

[7] Turer CC, Durmus D, Balli U, Guven B (2017). Effect of non-surgical periodontal treatment on gingival crevicular fluid and serum endocan, vascular endothelial growth factor-A, and tumor necrosis factor-alpha levels. J Periodontol.

[8] Al-Ghurabi B H (2021). The Role of Soluble TLR-2 in the Immunopathogenesis of Gingivitis. Internat Med.

[9] Karki R, Kanneganti T-D (2019). Role of AIM2 inflammasome in inflammatory diseases, cancer and infection. Eur J Immunol.

[10] Marchesan JT, Girnary MS, Moss K, Monaghan ET, Egnatz GJ, Jiao Y (2020). Role of inflammasomes in the pathogenesis of periodontal disease and therapeutics. Periodontol 2000.

[11] Jefferis BJ, Papacosta O, Owen CG, Wannamethee SG, Humphries SE, Woodward M (2011). Interleukin 18 and coronary heart disease: Prospective study and systematic review. Atherosclerosis.

[12] Weng Y, Ye B, Lin J, Lin S, Zhong L, Huang W (2022). Elevated circulating levels of gasdermin D are related to acute myocardial infarction and pyrogptosis. BMC Cardiovasc Disord.

[13] Sharma S, Mudgal S, Thakur K, Gaur R (2020). How to calculate sample size for observational and experimental nursing research studies?. Natl J Physiol Pharm Pharmacol.

[14] Tonetti MS, Greenwell H, Kornman KS (2018). Staging and grading of periodontitis: Framework and proposal of a new classification and case definition. J Periodontol.

[15] Chapple ILC, Mealey BL, Van Dyke TE, Bartold PM, Dommisch H, Eickholz P (2018). Periodontal health and gingival diseases and conditions on an intact and a reduced periodontium: Consensus report of workgroup 1 of the 2017 World Workshop on the Classification of Periodontal and Peri-Implant Diseases and Conditions. Clin Periodontol.

[16] Al-Taweel FBH, Saliem SS, Abd OH, Whawell SA (2021). Assessment of Serum Interleukin-1β and Interleukin-6 Levels in Patients with Chronic Periodontitis and Coronary Heart Disease. Eur J Gen Dent.

[17] Gita BJ, George AV, Pavithra N, Chandrasekaran S, Latchumanadhas K, Gnanamani A (2019). Dysregulation Of miR-146a by Periodontal Pathogens: a risk for Acute Coronary Syndrome. J Periodontol.

[18] Yagnik K, Mahendra J, Kurian VM (2019). The Periodontal-Cardiovascular alliance: Evaluation of miRNA-146a in subgingival plaque samples of chronic periodontitis patients with and without coronary heart disease. J Invest Clin Dent.

[19] Temelli B, Yetkin Z, Savas H, Aksoy F, Kumbul D, Uskun E, Varol E (2018). Circulation levels of acute phase proteins pentraxin 3 and serum amyloid A in atherosclerosis have correlations with periodontal inflamed surface area. J Appl Oral Sci.

[20] O'Leary TJ, Drake RB, Naylor JE (1972). The plaque control record. J Periodontol.

[21] Mühlemann HR, Son S (1971). Gingival sulcus bleeding–a leading symptom in initial gingivitis. Helv Odontol Acta.

[22] Cheng R, Wu Z, Li M, Shao M, Hu T (2020). Interleukin-1β is a potential therapeutic target for periodontitis: a narrative review. Int J Oral Sci.

[23] Ruwanpura SM, Noguchi K, Ishikawa I (2004). Prostaglandin E2 regulates interleukin-1beta-induced matrix metalloproteinase-3 production in human gingival fibroblasts. J Dent Res.

[24] Zardawi F, Gul S, Abdulkareem A, Sha A, Yates J (2021). Association Between Periodontal Disease and Atherosclerotic Cardiovascular Diseases: Revisited. Front Cardiovasc Med.

[25] Zhang C, Li H, Zhou G, Zhang Q, Zhang T, Li J (2007). Transcriptional silencing of the TMS1/ASC tumor suppressor gene by an epigenetic mechanism in hepatocellular carcinoma cells. J Pathol.

[26] Zhuang J, Wang Y, Qu F, Wu Y, Zhao D, Xu C (2019). Gasdermin-d Played a Critical Role in the Cyclic Stretch–Induced Inflammatory Reaction in Human Periodontal Ligament Cells. Inflammation.

[27] Shi H, Gao Y, Dong Z, Yang J, Gao R, Li X, Zhang S (2021). GSDMD-Mediated Cardiomyocyte Pyroptosis Promotes Myocardial I/R Injury. Circ Res.

[28] Al-Ghurabi BH, Mohssen SM (2015). Salivary level of RANKL and OPG in chronic periodontitis. J Bagh College Dentistry.

[29] Shaker ZF, Hashem BH (2012). Study the role of proinflammatory and anti-inflammatory cytokines in Iraqi chronic periodontitis patients. J Bagh College Dentistry.

[30] Genco RJ, Grossi SG, Ho A, Nishimura F, Murayama Y (2005). A proposed model linking inflammation to obesity, diabetes, and periodontal infections. J Periodontol.

